# Annotations

**Published:** 1894-05-12

**Authors:** 


### Annotations.

### Opium or Alcohol?

Assistant Surgeon T. M. Shah, Chief Medical Officer of the Junagadh State, a native with an European medical education, has written a most interesting article in the February number of the *Indian Medico-Chirurgical Review* on the use of opium in India. Among other questions, Surgeon Shah asks whether the native races of India would be advantaged by the substitution of alcohol for opium? His reply is in the following terms: "Alcohol exercises the most injurious influence on the moral and physical condition of the people of India. Its injurious effects on the body, and particularly in regard to crime, are increasing, and will culminate in future, as the use of alcohol is daily increasing. It is supplanting the use of both bháng and opium in India. Europe and other liquor-using countries (*sic*) are deploring the evil effects of it, particularly from a criminal point of view, and India will have at no distant date to send up the same cry to heaven. It is most striking to an impartial observer that no due attention is paid to alcohol, the king of evils, while such a hot war is waged against the humble, the innocent, and the honest opium." The tropes are a little Oriental, but there is no doubt of the honesty and intelligence of the writer. That opium may be and is misused, that many evils arise from its misuse, that legislation may with propriety control its culture and sale, Surgeon Shah admits. On the other hand he says that its moderate use by natives is not only quite harmless but often beneficial; that it is comforting and stimulating, both to body and mind; that it is of all stimulants the most wholesome, and the least liable to abuse; and that the natives of India could in no wise be deprived of it without serious public and private mischief and suffering. He admits that if all human beings were freed from cares and troubles, it might be allowable to prohibit stimulants of all kinds. But in a world like ours, of suffering and sorrow, he stands for stimulants; and of all stimulants opium for the Oriental is beyond all comparison the best.

### Preventive Family Practice.

Dr. William Ogle, of Derby, who has retired from practice, has not retired from a lively interest in the well-being of his professional brethren. This is all the more to Dr. Ogle's honour, in that as former physician and consulting physician to the Derby Infirmary he can never have known the *res augusta domi* which are but too familiar to many of the hard working and poorly paid general practitioners of the present day. Dr. Ogle has reissued in pamphlet form an excellent address on the preventive and general arrangements which family practitioners might advantageously make with their clients, an address delivered before the annual meeting of the British Medical Association so long ago as 1877. In a few telling points Dr. Ogle gives us to understand that medical men do far too much purely gratuitous work; that of preventive work they do too little; that preventive work is the very work of which they should do most, and for which they should be best paid; that the public do not pay willingly for preventive work, but only for "bottles" and "visits"; that this, however, is not the fault of the public, but of the medical profession, which has not educated the public; that it is all very unscientific and undignified, besides keeping the doctor in poverty and killing him with hard work; and that it ought to be ended without delay. Dr. Ogle's positions and arguments we believe to be all as sound and convincing as possible. Nevertheless, it was so long ago as 1877 that he first took up his parable; and here is the profession in 1894 not only no "forrarder," but deeper in the mire of poverty and slavery than before. If any man in the world is entitled to say to his professional brethren, "I told you so," that man is Dr. Ogle. The pamphlet suggests remedies, and good ones. We have not space to detail them; but Dr. Ogle, we believe, has a supply of copies ready for gratuitous distribution; and we wish every member of the profession, great and small, would send for one, and study it.

### Judges and Justice: Anderson v. Cook.

The jury have decided in this case that "the defendant," Mr. Justice Cook, "acted oppressively, and either maliciously or unaccountably under the influence of drink, overstrained his judicial powers to the prejudice of the plaintiff and for the perversion of justice." Ultimately they asked to be allowed to withdraw the reference to "drink"; but in all other respects their verdict, as stated, was confirmed. On the question of damages, a juror asked "why they might not give exemplary damages indicating their feelings as to the wrongs done to Dr. Anderson, even though the defendant might be unable to pay them?" In the result the damages were assessed at £500. The judge, on the other hand, gave judgment for the defendant on the ground that an action cannot lie against a judge. Let us note one or two utterances in Lord Coleridge's deliverance, which seem to indicate the trend of his mind in such cases. He expressed the opinion that the Tobago judges might have had provocation. Provocation! What would be thought of a doctor who because his patient was peevish and unreasonable, should order him a mixture injurious to his health? In a very airy way his lordship uttered these remarkable words, which we quote from the report in the *Times*, "Still, it was important that justice should be done to suitors." Important that justice be done to suitors—important! Why this is the very soul and essence of the whole business! What is law? A mere instrument for the discovering and the doing of justice. What are judges and lawyers? Mere servants, whom the State employs to use the instrument of the law for the one sole and only purpose of discovering and doing justice. In this case Dr. Anderson has in his own person represented outraged justice, according to the decision of the jury: the Tobago judges, on their part, have turned the law into an instrument, not of justice, but of oppression; and here we have the extraordinary spectacle of a Lord Chief Justice of England making, not a quasi, but an actual apology for the legal perverters of justice, and admitting, with apparent reluctance, that it is "important"—not absolutely essential, but "important that justice be done to suitors." We speak from the point of view of the Englishman, not of the doctor. It does not concern us at all in a case of this kind whether a man be a doctor or a chimney sweeper. The thing that does concern us, and every other responsible citizen of Great Britain, is the incomprehensible attitude of the chief representative of the majesty of the English law,

